# Podocin and uPAR are good biomarkers in cases of Focal and segmental glomerulosclerosis in pediatric renal biopsies

**DOI:** 10.1371/journal.pone.0217569

**Published:** 2019-06-12

**Authors:** Lívia Helena de Morais Pereira, Crislaine Aparecida da Silva, Maria Luíza Gonçalves dos Reis Monteiro, Liliane Silvano Araújo, Laura Penna Rocha, Marcelo Bernardes da Rocha Reis, Fernando Silva Ramalho, Rosana Rosa Miranda Corrêa, Marcos Vinicius Silva, Marlene Antonia Reis, Juliana Reis Machado

**Affiliations:** 1 Institute of Biological and Natural Sciences, Discipline of General Pathology, Federal University of Triangulo Mineiro, Uberaba, Minas Gerais, Brazil; 2 Department of Pathology and Forensic Medicine, Ribeirão Preto Faculty of Medicine of São Paulo University, Ribeirão Preto, São Paulo, Brazil; 3 Institute of Biological and Natural Sciences, Discipline of Parasitology, Federal University of Triangulo Mineiro, Uberaba, Minas Gerais, Brazil; University of Houston, UNITED STATES

## Abstract

There are controversies whether Minimal Change Disease (MCD) and Focal and Segmental Glomerulosclerosis (FSGS) are distinct glomerular lesions or different manifestations within the same spectrum of diseases. The uPAR (urokinase-type plasminogen activator receptor) and some slit diaphragm proteins may be altered in FSGS glomeruli and may function as biomarkers of the disease in renal biopsies. Thus, this study aims to evaluate the diagnostic potential of uPAR and glomerular proteins for differentiation between MCD and FSGS in renal pediatric biopsy. Renal biopsies from 50 children between 2 and 18 years old were selected, with diagnosis of MCD (n = 29) and FSGS (n = 21). Control group consisted of pediatric autopsies (n = 15) from patients younger than 18 years old, with no evidences of renal dysfunction. *In situ* expressions of WT1, nephrin, podocin and uPAR were evaluated by immunoperoxidase technique. Renal biopsy of patients with MCD and FSGS expressed fewer WT1 (p≤0.0001, F = 19.35) and nephrin (p<0.0001; H = 21.54) than patients in the control group. FSGS patients expressed fewer podocin than control (p<0.0359, H = 6.655). FSGS cases expressed more uPAR than each of control and MCD (p = 0.0019; H = 12.57) and there was a positive and significant correlation between nephrin and podocin (p = 0.0026, rS = 0.6502) in these cases. Podocin had sensitivity of 73.3% and specificity of 86.7% (p = 0.0068) and uPAR had sensitivity of 78.9% and specificity of 73.3% (p = 0.0040) for diagnosis of FSGS patients. The main limitation of the study is the limited number of cases due to the difficulty in performing biopsy in pediatric patients. Podocin and uPAR are good markers for FSGS and differentiate these cases from MCD, reinforcing the theory of distinct glomerular diseases. These findings suggest that podocin and uPAR can be used as biomarkers in the routine analysis of renal biopsies in cases of podocytopathies when the lesion (sclerosis) is not sampled.

## Introduction

Nephrotic syndrome (NS) in children comprises a diverse group of diseases, including podocytopathies of genetic causes with early onset or developed idiopathically. Minimal Change Disease (MCD) and Focal Segmental Glomerulosclerosis (FSGS) are the main idiopathic podocytopathies reaching the pediatric age group [1]. They have a similar clinical presentation generally with nephrotic syndrome, due to the effacement of foot processes and alteration in proteins of the slit diaphragm (SD). In the early stages of the disease, MCD and FSGS show great similarity [2], however, they seem to have different development mechanisms [3]. There are controversies among researchers, as some believe they are the same disease at different evolutionary stages and others believe they are distinct diseases. However, in some cases, due to the sample representativeness of a renal biopsy, the findings may suggest MCD, but the biopsy performed in another occasion would show typical characteristics of FSGS [4, 5].

The SD is one of the key elements of glomerular filtration barrier, and its proteins appear to be altered in podocytopathies. Nephrin is the main and the most abundant protein of SD, has intracellular, transmembrane and extracellular domains, the latter domain forms pores through interactions with each other, which have smaller diameters than albumin. Podocin is a membrane protein located exclusively in the SD region, with two intracellular domains that interact with nephrin. Changes in nephrin and podocin structures can lead to intense proteinuria [6–8].

In MCD, there are diffuse foot process effacement with conserved podocytes number, which keeps the glomerular structure. In FSGS, death of podocytes occurs and their detachment from glomerular basement membrane (GBM) leaves it naked and exposed to parietal cells in the Bowman's capsule. This exposure allows adhesion of the naked region to parietal epithelium with formation of synechiae, capillary lumen closure, and later sclerosis [9].

In idiopathic FSGS, which occurs in the absence of genetic defects, it has been hypothesized that a circulating factor would be the cause of the disease. Recently, the increased concentration of soluble urokinase-type plasminogen activator receptor (suPAR) has been proposed as a circulating risk factor specific for FSGS [3]. Evidences that support the existence of an etiological circulating factor are FSGS recurrence after transplantation and the clinical improvement of patients with FSGS when performing plasmapheresis [10].

Based on the challenges for diagnostic differentiation between FSGS and MCD, which may lead to delayed therapy, repeated biopsies and progressive impairment of renal function, the objective of this study was to evaluate the diagnostic potential of uPAR and podocyte proteins in differentiation between MCD and FSGS in pediatric renal biopsies.

## Materials and methods

### Patients

The study was approved by Ethics and Research Committee of the Federal University of Triangulo Mineiro (UFTM), with the number 1.715.838. All samples were archived and cases were identified by codes with letters and numbers to ensure that individuals were anonymized. Because it is a retrospective study, ethics committee waived the requirement for informed consent. Two hundred and seventy-four cases of pediatric renal biopsies between 1996 and 2015 were selected from the database of the Nephropathology Service of Federal University of Triângulo Mineiro. Of these, 50 were diagnosed as podocytopathies (FSGS and MCD). Renal biopsy indication in children were based on non-response to corticosteroid treatment, cases with nephrotic syndrome above 8 years-old, hematuria, persistent severe hypertension, reduced serum complement or loss of renal function [11]. Diagnosis were made in native kidney fragments with three samples for analysis by light microscopy, immunofluorescence and transmission electron microscopy (TEM). FSGS (21 cases) was diagnosed morphologically by the presence of focal and segmental sclerosis, followed by foot process effacement as seen by TEM, without other findings. MCD (29 cases) was diagnosed when the only morphological alteration was foot process effacement in TEM. The control group consisted of 15 renal samples of autopsied children, aged less than 18 years, with no evidences of infection and no previous renal changes. Cases with autolysis, acute tubular necrosis and congestion with moderate to severe alterations were also excluded from control group. Renal histological analysis of control group was performed by the same nephropathologist who evaluated podocytopathies cases. These samples were obtained from the Pediatric Pathology Service of the University of São Paulo / Ribeirão Preto.

### Immunoperoxidase technique

Tissue sections were 2 μm thick and were deparaffinized in xylol and hydrated in alcohol. Antigen retrieval was performed with Citrate buffer pH 6.0 (Bond Epitope Retrieval Solution 1, ready for use, Leica Biosystems) in Pascal pan (DAKO) and then, slides were washed with PBS buffer and incubated in a humid and dark chamber with peroxidase blocker (Peroxidase block, Kit Novolink Polymer Detection System, BL, UK) for 50 minutes. They were incubated in Protein Block solution (Protein block Kit Novolink Polymer Detection System, BL, UK) for 50 minutes. Then, slides were incubated with primary antibodies, anti-WT1 (1: 100; DAKO), anti-Nephrin (anti-NPHS1; 1:150; Abcam) and anti-Podocin (anti-NPHS2; 1:600; Abcam) for 2 hours at room temperature and anti-uPAR (1:50; Abcam) *overnight* in a humid and dark chamber at 4ºC. After that, slides were washed with PBS and incubated with the post-primary block reagent (Novolink Polymer Detection System, BL, UK) for 50 minutes at room temperature, washed with PBS and incubated with the polymer reagent (Novolink Polymer Detection System, BL, UK) for 50 minutes. Finally, they were washed with PBS and incubated with chromogen (Liquid DAB, Kit Novolink Polymer Detection System, BL, UK) for 1 minute for anti-NPHS1 and anti-NPHS2 and for 2 minutes for anti-WT1 and anti-uPAR. Subsequently, they were washed with running water and counterstained in Harris Hematoxylin (Neon Comercial) for 1 minute.

### *In situ* quantification

All fields containing a glomerulus were evaluated, with analysis of all glomeruli in renal biopsy slides and autopsy kidney fragments. For nephrin and podocin labeling, brownish staining was indicated by the observer using a semi-automatic interactive image analyzer system, Leica Qwin, in a 40x objective (1600X). Results were expressed as percentage of labeled area in relation to evaluated fields [12].

Immunostained cells in glomeruli were counted as WT1 or uPAR positive cells. These results were expressed as density of cells marked by total glomerular area. The result was expressed in cell density per glomerular area (cell/mm^2^), adapted from Sanden (2003) [13].

### Statistical analysis

Statistical analysis was performed in the program GraphPad Prism version 5.0. Normality was tested by Shapiro Wilk test. In cases of normal distribution and similar variances, ANOVA (F) parametric test followed by Tukey post-test and Student´s t test (t) were used. In cases with non-normal distribution, Kruskal-Wallis test (H) followed by Dunn post-test and Mann Whitney test (U) were used. In contingency tables analysis, Fisher's exact test was used (χ^2^). Correlation between two variables with non-normal distribution was analyzed by Spearman's test (rS). The diagnostic performance of biomarkers tested in renal biopsy was evaluated by sensitivity and specificity with the ROC curve (*Receiver Operating Characteristic*—ROC). Area under the curve (AUC), 95% confidence intervals (CI) and cutoff points were calculated using non-parametric methods. Differences were considered statistically significant when p < 0.05.

## Results

### Epidemiological and clinical profiles

Fifty cases of pediatric biopsies were selected, of which 26 were male (52%), 37 were white (74%) and median age was 12 (2–18) years. In patients with MCD, median age was 12 (2–17) years, females (n = 15; 51.72%) with white color was the most prevalent group among patients. Patients with FSGS also had median age of 12 (2–18) years, 12 patients were male (57%) and 15 were white (71.43%). Epidemiological profile of patients is detailed in Table 1.

**Table 1:** Epidemiological data on cases of MCD, FSGS and control group.

	Control (n = 15)	MCD (n = 29)	FSGS (n = 21)	Total of patients (n = 50)
**Age**				
*Mean ± SD*	7.4±4.3	10.6 ± 4.6	11.3 ± 4.9	10.9±4.7
*Median (Min-Max****)***	7 (2–14)	12 (2–17)	12 (2–18)	12 (2–18)
**Gender n (%)**				
*Male*	12 (80.0%)	14 (48.2%)	12 (57.1%)	26 (52.0%)
*Female*	3 (20.0%)	15 (51.7%)	9 (42.8%)	24 (48.0%)
**Color**				
*White*	NI	22 (75.8%)	15 (71.4%)	37 (74.0%)
*Non-White*	NI	4 (13.7%)	6 (28.5%)	10 (20.0%)

MCD, Minimal change disease; FSGS, Focal and segmental glomerulosclerosis; n, number of cases; NI, not informed

Four patients were hypertensive in each group (p = 0.69) and hematuria was present in 6 patients with MCD and 7 patients with FSGS (p = 0.54). Regarding the mean proteinuria, patients with MCD had 2.5 ± 2.861 g / 24h and patients with FSGS had 4.7 ± 3.975 g / 24h (p = 0.06). Patients with FSGS showed a more unfavorable laboratorial profile, as they had elevated serum creatinine, urea and total cholesterol levels and lower glomerular filtration rate, however there was no statistically significant difference between groups. The clinical profile of patients is detailed in Table 2.

**Table 2:** Clinical and laboratorial data of patients with FSGS and MCD.

	MCD (n = 29)	FSGS (n = 21)	P value and statistical tests
**Hypertension**			
*Yes*	4 (13.8%)	4 (19.0%)	
*No*	20 (69.0%)	12 (57.0%)	p = 0.69; χ^2^ = 0.73
*NI*	5 (17.2%)	5 (24.0%)	
**Hematuria**			
*Yes*	6 (21.0%)	7 (33.3%)	
*No*	13 (44.8%)	9 (42.9%)	p = 0.54; χ^2^ = 1.22
*NI*	10 (34.2%)	5(23.8%)	
**Proteinuria (g/24h)**	2.5±2.861	4.7±3.975	p = 0.06; U = 104.0
**Creatinine (mg/dL)**	0.6±0.2	2.1±4.2	p = 0.15; U = 88.0
**Urea (mg/dL)**	24.6±6.8	46.6±50.5	p = 0.05; U = 59.6
**Albumin (g/dL)**	2.8±1.1	2.96±1.0	p = 0.61, t = 0.51
**Total cholesterol (mg/dL)**	285.4±106.0	348.9±165.5	p = 0.15; t = 1.49
**eGFR (ml/min/1.73m**^**2**^**)**	128.0±80.0	102.2±95.3	p = 0.16; U = 42.0

MCD, Minimal change disease; FSGS, Focal and segmental glomerulosclerosis; n, number of cases; NI, not informed; U, Mann Whitney test; χ^2^, Fisher's exact test and t, Student´s t test

### Glomerular *in situ* expression of WT1, nephrin, podocin and uPAR

**Fig 1:**
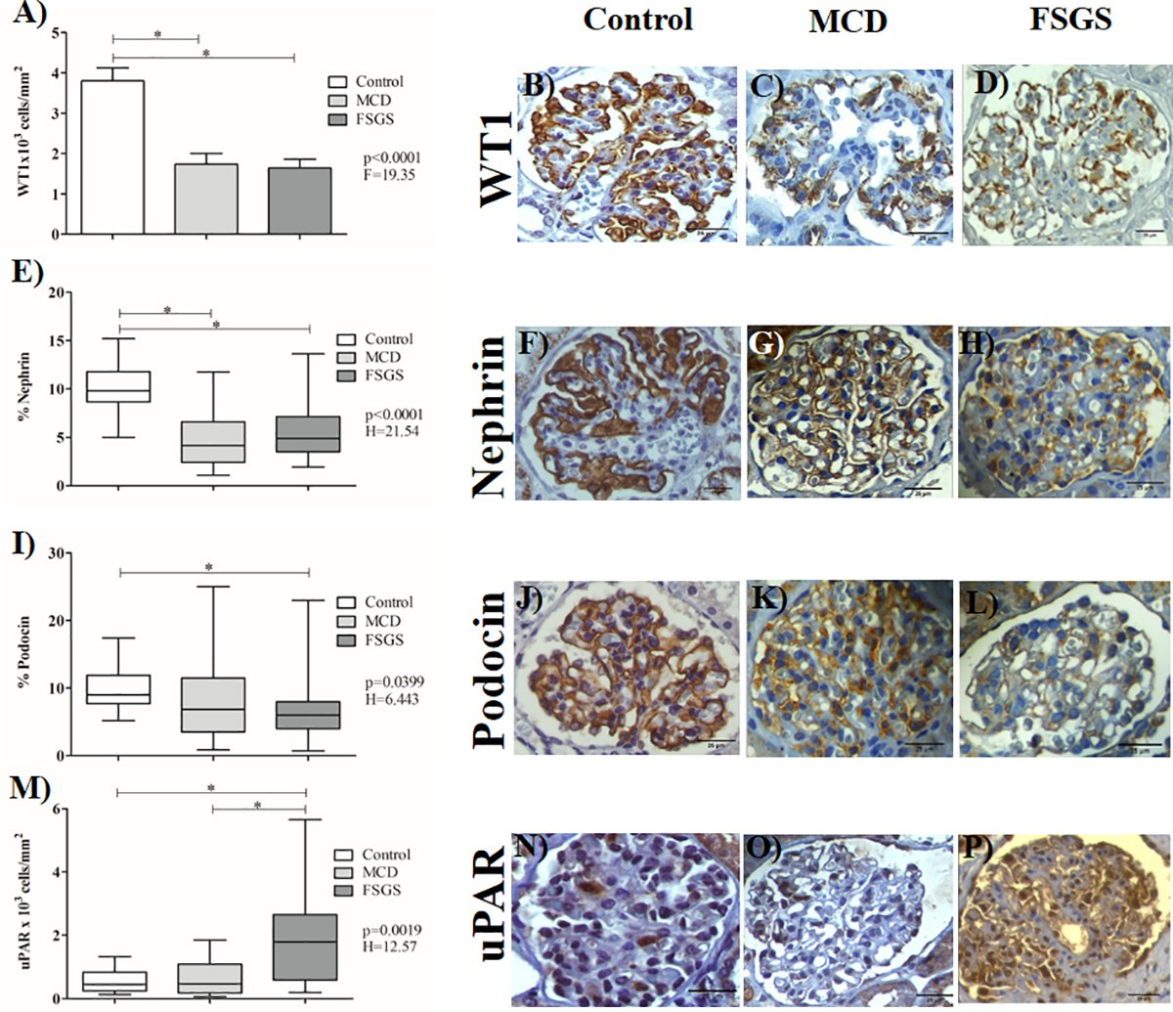
Expression of WT1, nephrin, podocin and uPAR in glomeruli by immunoperoxidase technique in the 3 groups: Control, MCD and FSGS. (A) Expression of WT1 in the 3 groups. ANOVA test followed by Tukey's multiple comparison test when normal distribution and bars represent the mean and the line above represents the standard deviation. WT1 immunolabeling in glomeruli in control (B), MCD (C) and FSGS groups (D) (1600X). (E, I and M) Expression of nephrin, podocin and uPAR in the 3 groups. Kruskal-Wallis test followed by Dunn's multiple comparison test when non-normal distribution and the horizontal lines represent the medians, the bars represent the 25–75% percentiles and the vertical lines represent the percentiles 10–90%. Nephrin immunolabeling in glomeruli in control (F), MCD (G) and FSGS (H) groups (1600X). Podocin immunolabeling in glomeruli in control (J), MCD (K) and FSGS groups (L) (1600X). uPAR immunolabeling in glomerular compartment in control (N), MCD (O) and FSGS groups (P) (1600X). *Statistical significance was defined as p<0.05.

Renal biopsies of patients with podocytopathies, MCD or FSGS, had lower WT1 immunolabeling, which means lower number of podocytes compared to control group (p≤0.0001, F = 19.35, Tukey's post test, Fig 1A to 1D). Similarly, renal biopsies of patients with MCD or FSGS presented decreased nephrin immunolabeling compared to control group (p<0.0001; H = 21.54, Dunn´s post test, Fig 1E to 1H). Renal biopsies of FSGS patients showed decreased podocin immunolabeling compared to control group. No difference was observed between MCD patients and control group regarding podocin staining (p<0.0359; H = 6.655, Dunn´s post test, Fig 1I to 1L). FSGS patients showed greater uPAR immunoexpression compared to patients in control and MCD groups (p = 0.0019; H = 12.57, Dunn´s post test, Fig 1M to 1P).

### Analysis of biomarkers correlations

**Fig 2:**
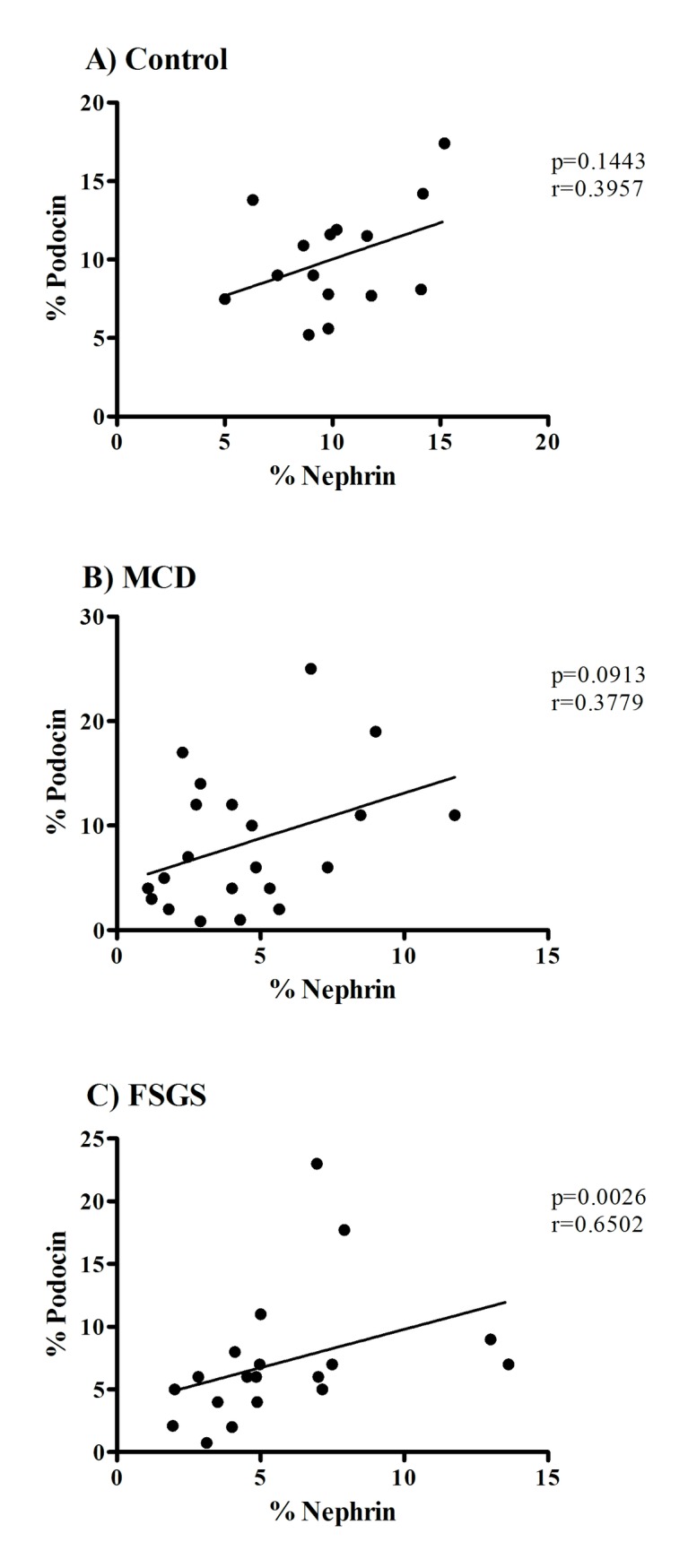
Correlation of nephrin and podocin expression in patients of control, MCD and FSGS groups. Correlation between (A) percentage in nephrin area and percentage in podocin area in control patients, (B) percentage in nephrin area and percentage in podocin area in patients in MCD group, (C) percentage in nephrin area and percentage in podocin area in patients of FSGS group. Used Spearman’s correlation test (rS) and Pearson’s correlation test (r). *Statistical significance was defined as p <0.05.

No correlation was observed between these proteins in control group and in MCD patients (Fig 2A and 2B). In patients with FSGS, a positive and significant correlation between nephrin and podocin was observed (p = 0.0026, rS = 0.6502, Fig 2C).

### Sensitivity and specificity analysis

Considering the lower expression of podocin and higher expression of uPAR in FSGS group, ROC curve was used to predict the potential of these proteins in differential diagnosis between MCD and FSGS.

**Fig 3:**
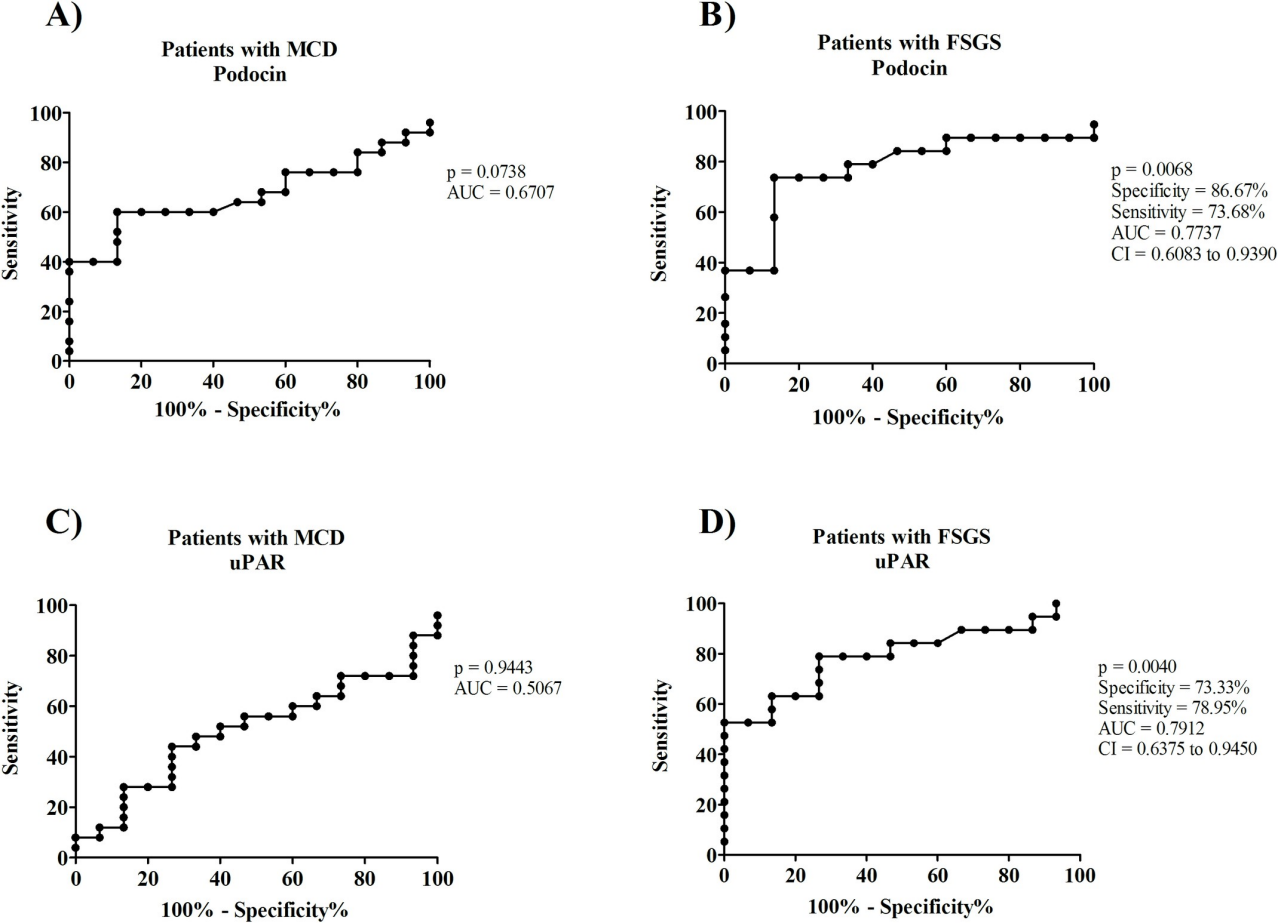
The receiver operating curves for models predicting podocin and uPAR performance for diagnosis of MCD and FSGS. (A) ROC curve graphic of plotted sensibility and 1-specificity of Podocin for MCD diagnosis. (B) ROC curve graphic of plotted sensibility and 1-specificity of Podocin for FSGS diagnosis. (C) ROC curve graphic of plotted sensibility and 1-specificity of uPAR for MCD diagnosis. (D) ROC curve graphic of plotted sensibility and 1-specificity of uPAR for FSGS diagnosis.

Podocin AUC in MCD was 0.6707 (IC_95%_ 0.5046–0.8368), with a cutoff point of 7.750, the sensitivity was 60,00%, and the specificity was 73,33% (p = 0.0738, Fig 3A). In FSGS, podocin AUC was 0.7737 (IC_95%_ 0.6083–0.9390), cut-off point of 7.24, sensitivity of 73.38% and specificity of 86.67% (p = 0.0068, Fig 3B).

Regarding uPAR, MCD AUC was 0.5067 (IC_95%_ 0.3256–0.6877), with a cutoff point of 0.48, sensitivity of 48.00% and specificity of 66.67% (p = 0.9443, Fig 3C). In FSGS, AUC was 0.7912 (IC_95%_ 0.6375–0.9450), cutoff point was 0.54, sensitivity was 78.95%, and specificity was 73.33% (p = 0.0040, Fig 3D).

## Discussion

The relationship between MCD and FSGS is controversial, as there are still doubts whether they represent distinct diseases or if they are only at different evolutionary stages. The morphological diagnosis of these two podocytopathies is a challenge for the nephropathologist, especially when biopsies do not contain adequate number of glomeruli and / or segmental sclerosis is not sampled. In this way, establishing specific biomarkers to differentiate these entities is of paramount importance.

In 2008, it was demonstrated for the first time that uPAR activation in podocytes was associated with FSGS in humans [14]. In 2014, Smeets et al used the detection of activated parietal epithelial cells with the objective of identifying a cellular marker that contributed to the distinction between initial FSGS and DCM [15].

Thus, in this study, we evaluated the sensitivity and specificity of uPAR and some podocyte proteins as potential biomarkers in differential of MCD and FSGS by immunostaining in renal biopsy.

In childhood renal diseases, nephrotic syndrome is the most common clinical manifestation and in about 90% of cases, it involves primary glomerulopathy. The main diagnosis for childhood nephrotic syndrome is MCD, comprising about 90% of the cases, followed by FSGS and diffuse mesangial proliferation [16–18].

MCD and FSGS can be classified as podocytopathies as the morphology and function of podocytes are disrupted. One way to assess podocyte density is through renal biopsy. As suggested by other researchers, WT1 has diagnostic utility, because during nephrogenesis it is expressed in glomerular progenitor cells and helps in renal development. As glomerular maturation proceeds, WT1 becomes restricted to podocyte nuclei and plays an important role in its homeostasis, which makes this protein important in identifying loss of these cells [19].

In the present study, we found a reduction of this protein in patients with MCD and FSGS compared to control group. Animal studies with induction of glomerulosclerosis showed a decrease in podocytes number in addition to an increase in glomerular volume. This reduction could have been due to podocytes apoptosis, detachment from the GBM and consequent release in urine [20]. The remaining podocytes would undergo a "stretching" process on GBM to compensate the absence of those that stood out and this process is responsible for activating Angiotensin II and Angiotensin I receptors, which are inducers of apoptosis via TGF-β [21]. This mechanism could explain the reduction in podocytes number in our cases of FSGS. However, in cases of MCD, podocyte number was expected to be maintained. However, we found a reduction in podocyte number compared to control group. It is believed that there is no real reduction in podocytes number, but a defect in the transcription of WT1 protein, due to a podocyte phenotypic change, which would make WT1 expression reduced [20].

Nephrin expression was decreased in patients with MCD and FSGS compared to patients in control group. SD proteins are of great importance in keeping the filtration barrier while maintaining control of podocyte structure and signaling pathways. Nephrin has an important role in the structure of this diaphragm, being its main component and having several interactions with other proteins that help keeping the structure of the filtration barrier. Glomerular diseases with proteinuria and foot process effacement have changes in expression and location of SD proteins [22]. Researchers found reduced nephrin immunoexpression in MCD compared to control cases and, in addition, very high levels of proteinuria [22]. Studies on nephrin expression by immunofluorescence showed a huge loss of immunostaining pattern regardless of whether the disease was primary, acquired or congenital, and also identified that this loss was not different between types of glomerulopathies such as MCD, FSGS or membranous glomerulopathy [23]. Some studies found no difference in nephrin expression in cases of MCD compared to FSGS using *in situ* hybridization and immunohistochemistry [23, 24]. However, studies using rats with glomerulosclerosis evaluated the structure of SD and found that both areas of sclerosis and without sclerosis showed nephrin reduction. Thus, glomerular sclerosis was responsible for nephrin loss due to podocytes detachment and subsequent SD disruption. Moreover, in cases without sclerosis, the explanation would be SD proteins redistribution due to changes in nephrin expression and fragmentation of its quantification [20]. It is possible that reduced nephrin expression is not related to a specific glomerular disease, but rather to the state of proteinuria, a common denominator among the cases discussed, and that nephrin is the target of these elevated levels of protein [25].

In this study, FSGS patients had reduced podocin compared to control group. Podocin is a membrane protein expressed exclusively in podocytes. Mutations in NPHS2 gene encoding podocin are found in up to one-third of pediatric patients with steroid resistant nephrotic syndrome [26–30]. Explanation for the reduction or complete absence of podocin in cases of idiopathic nephrotic syndrome still requires many studies, but it is known that reduction in podocin expression as well as nephrin is involved in podocytes phenotypic changes [29].

A study conducted in Japan analyzed podocin expression in different glomerular diseases. In MCD, no podocin reduction was found compared to control cases and all had a satisfactory response to corticosteroid therapy. In contrast, in FSGS patients, 74% had reduction or absence of podocin expression and a poor response to corticosteroid treatment, and some patients progressed to end-stage renal disease. In addition, little or no reduction in podocin expression was found in diseases such as IgA Nephropathy, Membranous Glomerulopathy and Henoch-Schönlein Purpura Nephritis [31]. In another study with renal biopsies using immunofluorescence and immunoperoxidase labeling, a reduction in podocin expression was found in up to 90% of FSGS cases [29]. Thus, it is possible to infer that podocin seems to be an important SD protein in the differentiation of FSGS from other glomerulopathies, especially MCD, which sometimes makes a differential diagnosis with FSGS. There was a positive and significant correlation between nephrin and podocin in FSGS. Podocin can interact with CD2AP and especially with nephrin, suggesting a function that regulates the dynamics of the actin cytoskeleton, responsible for anchoring SD [29, 31, 32]. In FSGS, the process of mesangial matrix expansion, death of podocytes with posterior detachment and exposure of GBM [9], leads to destruction of SD composition, including its major proteins, nephrin and podocin, and hence its direct correlation in FSGS.

Patients with FSGS showed greater uPAR expression in glomeruli compared to MCD and control group. FSGS can account for about 20% of glomerular diseases and, on average, 80% of the cases are idiopathic. In many situations, it has a poor clinical course evolving to dialysis and transplantation. The rate of disease recurrence after transplantation is high in both adults and children, with rapid onset of lesions in the graft. This has been a strong indication that there is a circulating factor that participates in disease development [3]. Other findings supporting the involvement of a circulating factor include reduction of proteinuria in response to plasmapheresis [33] or immunoadsorption [34], and one case of transient nephrotic syndrome in a newborn whose mother had FSGS [35].

Some specialists argue that uPAR would act in podocytes and have a role in triggering glomerular diseases [3, 36–38]. uPAR is a glycosylphosphatidylinositol anchored in 3 protein domains (DI, DII and DIII), which acts as a cellular receptor of urokinase and participates in signaling through cellular and other transmembrane receptors, such as integrins. It is expressed on the cell surface of several cell types. uPAR can be cleaved in its domains by proteolytic enzymes or by the action of phospholipases and become soluble in the circulation (suPAR). Low levels of suPAR are physiologically available in human circulation as it is derived from hematopoietic, endothelial cells, fibroblasts and smooth muscle cells [3, 39]. In addition, uPAR is also involved in many other non-proteolytic biological processes, such as migration, adhesion, angiogenesis and cell proliferation [40].

The theory about the development of FSGS would be the existence of a circulating factor. However, the circulating agent suPAR, comes from a membrane bound factor, uPAR, which upon activation by its binder allows cleavage in the soluble form [3, 37]. Our study in renal biopsies analyzed the cell membrane-bound form in glomeruli using an specific rabbit polyclonal antibody to uPAR. An experimental study found elevated levels of suPAR in the circulation of uPA *knockout* mice induced to the development of FSGS and proteinuria. This indicates that, even without the uPAR binder, its cleavage occurred and suPAR was formed. There seem to be other molecules binding to uPAR, which promote its activation, cleavage and release in the circulation [41]. Its mechanism of action is associated with binding and activation of β3 integrin in podocyte membrane. Both uPAR and suPAR seem to have the ability to activate αvβ3 integrin and thus promote cell motility and activation of small GTPases, such as Cdc42 and Rac1, which can lead to podocyte contraction, shift from a stationary to a motile phenotype and lead to foot process effacement [42]. This activation by circulating systemic suPAR depends on its individual serum amount [3] and can also be driven by augmented podocyte uPAR expression, which is sufficient to initiate podocyte foot process effacement and proteinuria [14].

Studies with urinary analysis of suPAR [43], as well as serum analysis [36, 38] found much higher levels of this marker in FSGS than in other diseases, such as Diabetic Nephropathy, IgA Nephropathy, Membranous Glomerulopathy and MCD. In addition, it was correlated with lower values of glomerular filtration rate and development of proteinuria [44]. Thus, comparing with MCD, FSGS levels were much higher and uPAR could help in diagnostic differentiation between these two diseases in histopathological analysis.

A study that evaluated an immortalized podocyte cell line showed that exposition to suPAR triggered an increase of TRPC6 channels on podocyte surface and a reduction of podocin. Podocin decreased even more when podocytes were exposed to suPAR along with TNF. Cells exposed to serum and plasma from patients with recurrent FSGS had very low levels of podocin. The mechanism leading to podocin reduction is uncertain but appears to be involved with the αvβ3 integrin activation pathway [45].

Due to the difference in biomarkers expression between groups and their proximity of location and function, we inquired a possible density correlation between them. In our study, the ROC curve analysis allowed identification of markers that could help distinguish between MCD and FSGS. According to sensitivity and specificity test, podocin and uPAR were highlighted as the two best targets for FSGS differentiation.

Podocyte depletion correlates directly with SD loss of nephrin and podocin proteins, culminating in changes in the glomerular filtration barrier. Podocin was selective as it was reduced only in patients with FSGS, constituting a strong indicator of the disease. Besides, it is involved in uPAR glomerular pathway of lesion. The mechanism we evidenced as a podocyte depletor in FSGS is the one triggered by uPAR, which seems to be a predictor and a possibly diagnostic marker of the disease, along with podocin.

Our data indicate that immunostaining for uPAR and podocin in pediatric renal biopsies has good potential to aid in the differentiation between FSGS and MCD. Based on our findings, we believe that the two-major childhood podocytopathies, MCD and FSGS, are distinct glomerular lesions and not patterns of the same disease at different stages. These findings, in consonance with some data in literature, encourage us to use podocin and uPAR as biomarkers in routine kidney biopsy analysis of cases of podocytopathies in which the lesion (sclerosis) is not sampled.

## Supporting information

S1 File Data in brief used to reach the conclusions drawn. (XLSX)
